# Establishment of sensitive sandwich−type chemiluminescence immunoassay for *Aspergillus* galactomannan antigen

**DOI:** 10.3389/fcimb.2025.1658070

**Published:** 2025-10-01

**Authors:** Junpu Li, Yan Guo

**Affiliations:** ^1^ The Clinical Laboratory, Tianjin Chest Hospital, Tianjin, China; ^2^ Department of Medical Ultrasound, Tianjin Medical University General Hospital, Tianjin, China

**Keywords:** galactomannan, immunoassay, chemiluminescent, serum, BALF

## Abstract

**Introduction:**

Invasive pulmonary aspergillosis (IPA) poses significant diagnostic challenges in immunocompromised patients, with galactomannan (GM) detection serving as a key biomarker. Existing Platelia™ GM enzyme-linked immunosorbent assay (ELISA) faces limitations in throughput and turnaround time.

**Methods:**

We developed a magnetic bead-based chemiluminescent enzyme immunoassay (CLEIA) for GM detection in serum/bronchoalveolar lavage fluid (BALF).

**Results:**

Optimized CLEIA parameters enabled 30-min analysis versus 120-min for ELISA. Validation with 241 clinical specimens demonstrated robust analytical performance: diagnostic thresholds of ≥ 0.20 CLEIA Units for serum and BALF matrices (ROC-AUC 0.87, p<0.0001), analytical sensitivity of 0.50 ng/mL, and precision with ≤14.40% total CV, and no hook effect ≤200 ng/mL. Method comparison against the Platelia ELISA revealed 86.72% overall diagnostic agreement (Cohen’s k=0.72), while cross-reactivity analyses indicated specificity limitations exclusively with *Histoplasma*-positive specimens, consistent with parallel GM ELISA results.

**Discussion:**

This assay enables 75% processing time reduction with uncompromised diagnostic accuracy, positioning it as a high-throughput alternative for rapid IA screening in high-risk cohorts.

## 1 Introduction

*Aspergillus* spp. are widespread filamentous fungi that thrive in diverse environments, both in indoor and outdoor settings (Hajjeh and Warnock, 2001; Fröhlich-Nowoisky et al., 2009; Arastehfar et al., 2021). Invasive pulmonary aspergillosis (IPA) is a severe infection caused by the *Aspergillus* fungus, predominantly affecting immunocompromised individuals, including patients receiving immunosuppressive therapies, organ transplant recipients, or those with advanced HIV/AIDS. Unlike mild forms of aspergillosis, the invasive type can rapidly disseminate from the pulmonary system to other organs such as the brain, kidneys, and cardiovascular system, posing a significant threat to patient survival (WHO fungal priority pathogens list to guide research, development and public health action, 2022; Denning, 2024; Schwarz et al., 2024; Herrera et al., 2025). Diagnosing this condition remains challenging, necessitating a comprehensive evaluation that integrates patient risk factors, clinical and radiological findings, and mycological evidence (Ostrosky-Zeichner, 2012; Donnelly et al., 2020). Early identification enables prompt initiation of antifungal therapy, reducing the mortality associated with this disease (Ambasta et al., 2015). Given its rapid progression, reliable diagnostic methods are essential for effectively managing the infection in high-risk populations.

Galactomannan (GM), a major component of the *Aspergillus* mold cell wall, is shed during fungal growth. Therefore, detecting this compound in blood or BALF serves as a valuable tool for diagnosing IPA (Miceli and Kauffman, 2017; Mercier et al., 2018). Traditional reference method for detecting GM antigen has been the Platelia enzyme-linked immunosorbent assay (ELISA) developed by Bio-Rad (Moura et al., 2018; Donnelly et al., 2020; Terrero-Salcedo and Powers-Fletcher, 2020). Nevertheless, it is still useful to develop innovative detection schemes for diagnostics. This technique is labor-intensive and complex, prompting the development of more efficient alternatives. Among these, chemiluminescence immunoassay has emerged as a highly promising approach due to its advantages, including simplified procedures, highly quick detection, a broad analytical range, superior sensitivity and specificity (Buil et al., 2023; Albert et al., 2024; Lo Cascio et al., 2024; Kılıç et al., 2025).

Here, we carried out the detection of *Aspergillus* GM antigen in serum and bronchoalveolar lavage fluid (BALF) samples using a sandwich chemiluminescence enzyme immunoassay (CLEIA). In this assay, the alkaline phosphatase (AP) was used as the signal marker, and magnetic beads (Mbs) were used as the solid carrier. The presence of target GM could cause a sandwiched immunoreaction between Mbs coated with GM polyclonal antibody (Mbs-Ab) and AP-labeled antibody (AP-Ab). The analytical parameters, which included the dilution ratio of Mbs-Ab, the concentration of AP-Ab, the incubation time, and the volume of APS solution were first optimized. Then, analytical performances such as the cutoff value, analytical sensitivity, high-dose hook effect, precision, interference, cross-reactivity, and method comparison were evaluated. Compared to existing GM CLEIA, our studies demonstrate that the CLEIA developed here offers distinct advantages: enhanced detection result stability (CV% <15%) and significantly faster turnaround times (30min vs >1h) (Calero et al., 2022).

## 2 Materials and methods

### 2.1 Reagents and instruments

The coating *Aspergillus* GM antibody was obtained from KeraFAST (Shanghai, China). The labeled *Aspergillus* GM antibody was acquired from ThermoFisher Scientific (Shanghai, China). GM antigen (purity, >95%) stock was provided by our laboratory. The magnetic beads (1 μm diameter) and the desalting column were from Thermo Fisher Scientific (Shanghai, China). N-(3-(dimethylamino)propyl)-N’-ethylcarbodiimide hydrochloride (EDC), N-hydroxysulfosuccinimide (NHS) and, 4-morpholineethanesulfonic acid (MES), bovine serum albumin (BSA) were purchased from Sigma-Aldrich (Shanghai, China). Alkaline phosphatase (AP) for labeling was purchased from Roche Diagnostics (Shanghai, China). APS-5 was bought by Lumigen (Michigan, USA). Other chemicals unless stated otherwise, were sourced from Solarbio (Beijing, China). All reagents were of analytical or higher grade. Ultrapure water (18.2 MΩ·cm) was made by a Millipore Milli-Q system. Ultraviolet absorption spectra were acquired using a NanoDrop 2000c system (ThermoFisher Scientific). The automated chemiluminescence analyzer (DF200) was provided by Danda Biotech ((Beijing, China).

### 2.2 Sample collection

Between February 2024 and June 2025, we enrolled a total of 241 participants at Tianjin Chest Hospital. The cohort comprised 86 patients with probable IPA classified according to the 2020 EORTC/MSG Criteria (mycologic evidence based on positive Platelia ELISA results, *Aspergillus* PCR detection or positive culture result), 90 patients without evidence of IPA, and 88 healthy volunteers. Participants had a median age of 45 years (range 21–86 years), with 108 (45%) being female. Medical records were available and reviewed for the 176 patients in the cohort (excluding healthy volunteers). Within this patient subgroup, clinical characteristics included underlying hematological malignancies in 58 patients (33%), solid organ transplantation in 35 patients (20%), ICU care requirement for various underlying diseases in 39 patients (22%), and a history of long-term corticosteroid or immunosuppressant use in most of the remaining patients. Patients meeting any of the following exclusion criteria were not included: 1) *Aspergillus* colonization or contamination; 2) Unavailability of required diagnostic markers. Based on recent CT scans, BALF was collected from targeted bronchial segments by instilling ~50 ml of room temperature saline, repeated 2–3 times. The fluid was collected sterilely and sent for prompt analysis (within 30 minutes). Both BALF and serum samples were collected within 24 hours of patient admission and prior to antifungal treatment. Of these samples, sera (n=131) and BALF (n=110) with sufficient volume remaining after routine clinical analyses were aliquoted and stored at -80 °C for future use. Repeated freeze-thaw cycles were strictly avoided. The study protocol and all study-related procedures was approved by the Ethics Committee of Tianjin Chest Hospital (Grant No: 2023YS-019-1) and a waiver of informed consent was granted.

### 2.3 Preparation of anti−GM antibodies coated with magnetic beads

GM antibody-functionalized MBs were prepared according to supplier provided protocols along with a laboratory-developed modification method. Briefly, 2 mg of Mbs were washed twice with deionized water using a magnetic separator. The carboxyl group on the surface of MBs were next activated in 1.0 mL of MES buffer solution (50 mM) containing 6 mg EDC and 4 mg NHS for 30min under a shaker. The activated MBs-COOH were purified via three MES buffer washes and resuspended in 0.5 mL of MES buffer. Following that, 100 μL of Ab (1 mg/mL) was added to the above suspension and allowed to react for 4h with gentle agitation, after which blocking was performed using 100 μL of BSA (10 mg/mL) for an additional 2h. Finally, the obtained MBs-Ab were washed, redispersed with 1 mL of PBS buffer containing 0.2 mg/mL BSA, and stored at 4°C for next use. Mbs-Ab formation was tracked using the Bicinchoninic acid (BCA) protein quantification method and the lack of residual antibody in the coupling buffer supernatant validated bioconjugation.

### 2.4 Preparation of anti−GM labeled antibodies−alkaline phosphatase

The GM antibodies and AP were coupled using glutaraldehyde. Firstly, 2 mg of AP was dissolved in 0.5 mL of antibody solution (2 mg/mL), followed by dilution to a final volume of 2 mL with ultrapure water and thorough mixing. Then, 100 μL of 5% w/v glutaraldehyde solution was slowly added to the mixture under continuous stirring for 2h in dark. Lastly, the labeled antibody mixture was dialyzed in phosphate-buffered saline (PBS, pH 7.2) (0.2 M Na_2_HPO_4_ and 0.2 M NaH_2_PO_4_) overnight and purified using desalting column. The resulting AP-conjugated GM antibody (AP-Ab) was mixed with an equivalent volume of glycerol, and stored at 4°C. UV spectral analysis of AP-Ab showed a shift toward shorter wavelengths compared to AP and anti-GM, verifying AP-antibody conjugation and its potential for diagnostic use.

### 2.5 Immunoassay procedure for the detection of GM

**Figure 1:**
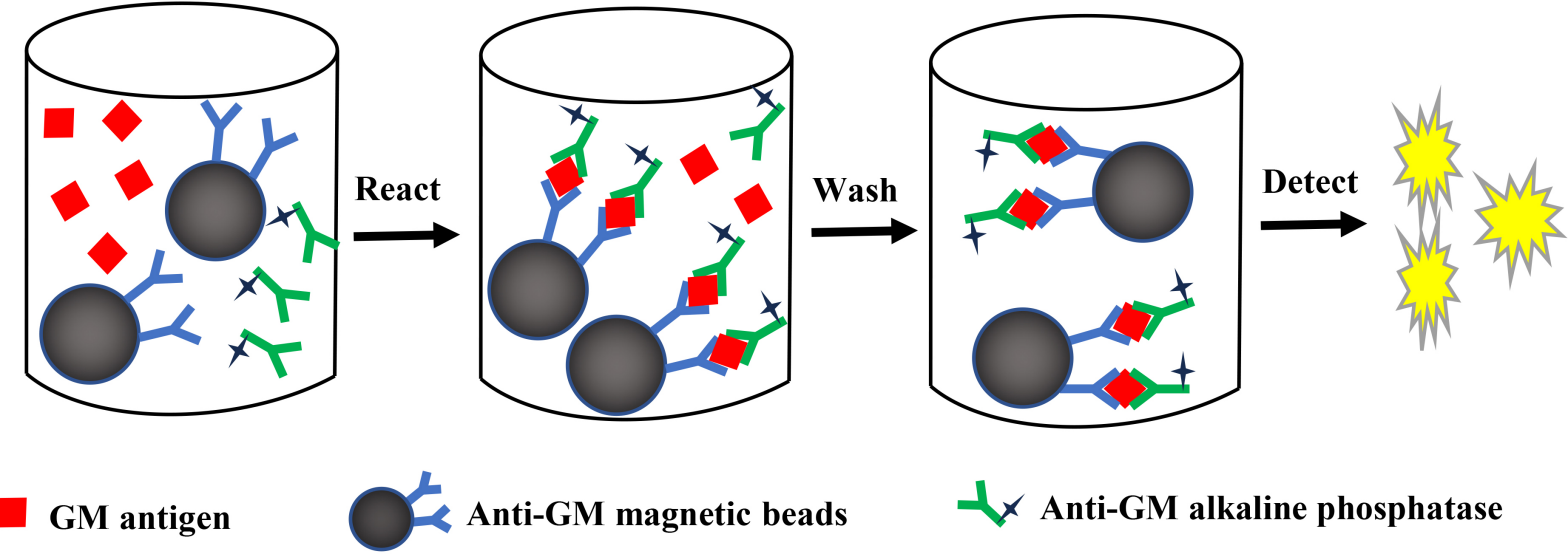
Schematic diagram of the detection process.

As shown in **Figure 1**, GM antigen was detected by sandwich immunoassay as follows. Prior to testing, all samples, including the calibrator control (2.5 ng/mL *Aspergillus* GM in BSA solution), serum, and BALF specimens underwent a 3:1 (v/v) dilution in 4% EDTA-PBS, followed by thermal denaturation (120°C, 5min) to eliminate protein interference. The treated samples were then centrifuged (10,000 × g, 5min) to remove precipitates. Equal volumes of anti-GM-AP (50 μL) and anti-GM-Mbs (50 μL) were added to 100 μL the above supernatant in reaction tubs. The obtained mixture was vortexed, incubated at 37°C for 20 mins, then subjected to three wash cycles via magnetic separation. Finally, the luminescent substrate solution (APS) was added to the sandwich Mbs-GM-AP immunocomplex, and the resulting chemiluminescent reaction was measured by the commercial chemiluminescence analyzer. The calculation of CLEIA Units was performed by dividing the chemiluminescent intensity of each sample by that of the calibrator control.

### 2.6 Statistical analysis

Duplicate measurements were performed for all test samples. The experimental results were statistically analyzed with the GraphPad Prism 9 software. Receiver operating characteristic (ROC) analysis was performed to establish an optimal positivity threshold and assess the overall diagnostic performance of CLEIA. Qualitative agreement between CLEIA and ELISA was assessed by calculating observed agreement and Cohen’s kappa statistic. Quantitative agreement was evaluated using Spearman’s rank correlation between the GM indices derived from both assays. Median values were compared using the Mann-Whitney U test. Repeatability was evaluated by the coefficient of variation (CV). A two-sided *p* value of less than 0.05 was considered to be statistically significant.

## 3 Result

### 3.1 Optimization of experimental conditions

The detection performance of the assay was found to depend critically on four parameters: the dilution ratio of Mbs-Ab, the concentration of AP-Ab, the incubation time, and the volume of APS solution. To optimize the testing conditions, the signal-to-noise (S/N) ratios derived from the two calibrators with the GM concentration of 1.0 ng/mL and 0 ng/mL were analyzed through the developed assay.

**Figure 2:**
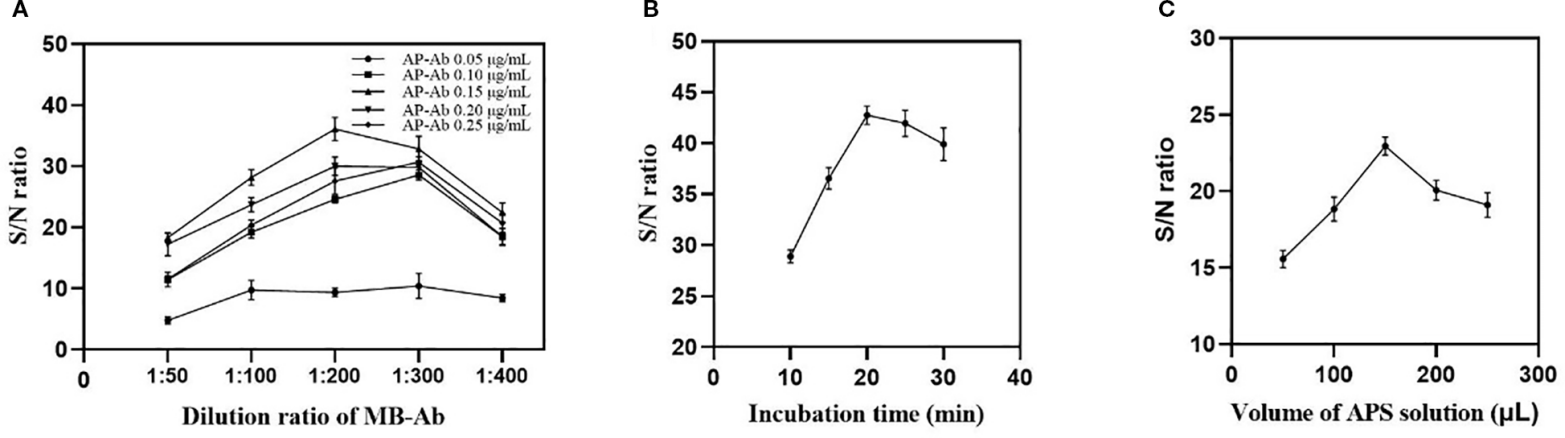
The optimization of **(A)** Mbs-Ab dilution ratios and AP-Ab concentration, **(B)** incubation times for the formation of sandwich immunoconjugates, **(C)** and volume of APS solution. Error bars represent standard deviations of three repetitive experiments (n = 3).

Initially, a checkerboard titration experiment was performed involving Mbs-Ab and AP-Ab. If the quantity of antibody conjugates is too low, it will be insufficient to capture all the target analytes present in the sample. Conversely, an excessive amount of these conjugates can interfere with luminescence or cause high background noise due to nonspecific interactions between the signal probe and the capture probe. Both scenarios would negatively impact the signal-to-noise ratio. As indicated by **Figure 2A**, the working parameters established were a 1:200 dilution for the Mbs-Ab and a concentration of 0.15 μg/mL for the AP-Ab.

Second, the incubation time for the immunoreaction between the antigen and antibody was also investigated. Analysis of incubation time (**Figure 2B**) revealed an initial S/N ratio increase over the first 20min, stabilizing (or marginally declining) between 20 and 30min. The maximum S/N was attained at 20min, confirming immunoreaction equilibrium. Considering both the absence of further S/N gains and the potential for increased nonspecific adsorption during prolonged incubation, 20min was chosen as the optimal reaction time.

Beyond the aforementioned reaction conditions, substrate volume critically influences luminescent signal amplitude and analytical sensitivity. Based on the data in **Figure 2C** showing a peak S/N ratio at 150 µL, this volume of APS was standardized for all subsequent experimental procedures.

### 3.2 Determination of the cut−off value

**Figure 3:**
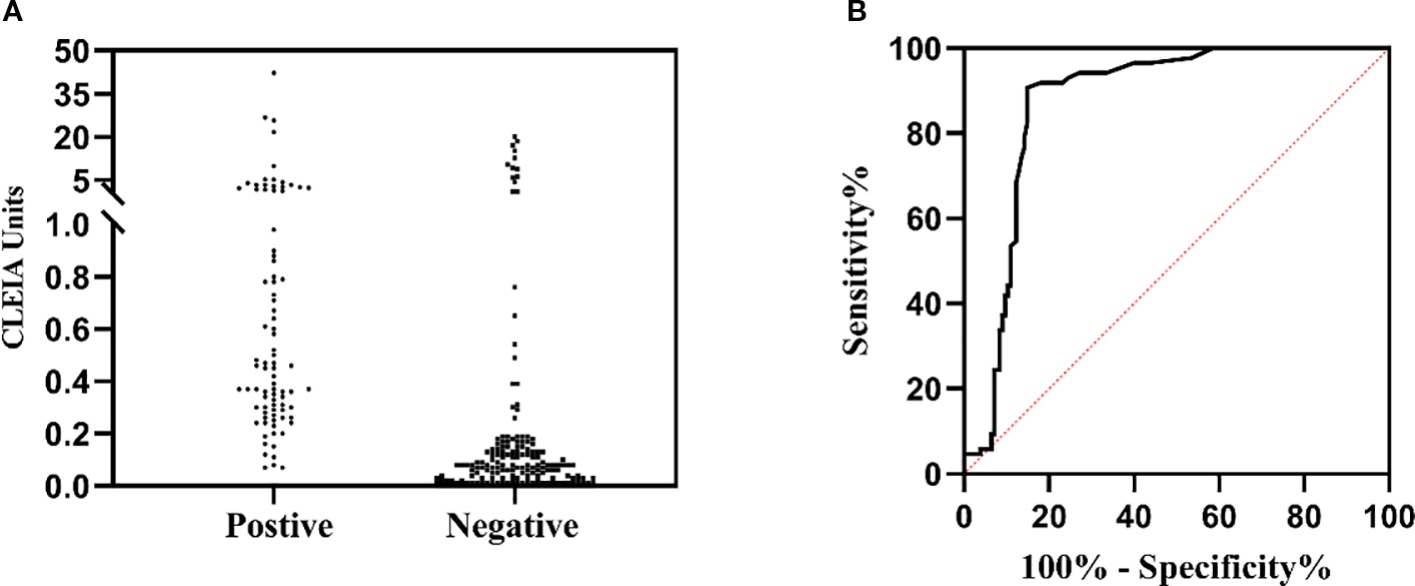
Determination of the cutoff value of the CLEIA for GM detection through ROC. **(A)** CLEIA Units measured in a specimen panel classified as positive or negative by the 2020 EORTC/MSG Criteria. **(B)** ROC curve derived from this analysis. The dotted line represents the cutoff value of our method.

To determine cut-off values, 241 retrospective specimens (131 serum, 110 BALF) from 241individuals were evaluated under the above optimal conditions. Analysis of GM antigen levels (CLEIA Units) revealed a range of 0.01-26.74 (**Figure 3A**), with statistically distinct clustering between positive and negative groups (*p*<0.0001). ROC curve evaluation (**Figure 3B**) determined an optimal cutoff value of 0.20 CLEIA Units, yielding diagnostic sensitivities of 90.70% (95% CI, 82.70 to 95.21) and specificities of 85.16% (95% CI, 78.72 to 89.90).

### 3.3 Analytical sensitivity (C_95_)

To assess CLEIA analytical sensitivity, we prepared serum and BALF samples spiked with *Aspergillus* GM antigen (4.00 ng/mL) followed by eight serial 1:2 dilutions. Testing included 20 replicates per concentration level. The analytical sensitivity—defined as the concentration achieving ≥95% positive results—was calculated to be 0.50 ng/mL for both matrices.

### 3.4 High dose hook effect

In sandwich immunoassays, the hook effect represents a critical analytical artifact wherein excessive analyte concentrations saturate both solid-phase and labeled antibodies. This antibody saturation prevents complete formation of ternary immune complexes, generating false-negative results.

As presented in **Table 1**, validation testing for hook effect revealed no false-negative CLEIA results in *Aspergillus* GM antigen-spiked human serum and BALF samples at concentrations up to 200 µg/mL.

**Table 1:** High-Dose hook effect results for GM detection.

Specimen	Sample concentration	Mean CLEIA Units	Result
BALF	250 µg/mL	0.11	Negative
	200 µg/mL	0.53	Positive
	150 µg/mL	1.68	Positive
	100 µg/mL	4.10	Positive
	50 µg/mL	4.76	Positive
Serum	250 µg/mL	0.16	Negative
	200 µg/mL	0.27	Positive
	150 µg/mL	0.52	Positive
	100 µg/mL	1.80	Positive
	50 µg/mL	2.24	Positive

### 3.5 Precision

Precision on the CLEIA was evaluated using a specimen panel comprising 4 pooled patient serum samples (negative, low positive, positive, high positive) and 4 pooled patient BALF samples spiked with purified GM (negative, high negative, low positive, positive). Each sample underwent 30 replicate measurements: two operators performed three independent runs per day over five consecutive days. The following metrics were calculated from CLEIA Units results to evaluate precision: mean, standard deviation (SD), percent coefficient of variation (%CV), percentage of positive identifications (% Positive), and percentage of negative identifications (% Negative). Comprehensive results are documented in **Table 2**, which indicated that the reproducibility and precision of this method are acceptable.

**Table 2:** Coefficient variations of the CLEIA from spiked specimens.

Sample type	Mean	SD	% CV	% Positive	% Negative
High Positive Serum	8.77	0.526	4.86	(30/30) 100%	(0/30) 0%
Positive BALF	0.68	0.050	7.35	(30/30) 100%	(0/30) 0%
Positive Serum	0.90	0.050	5.56	(30/30) 100%	(0/30) 0%
Low Positive BALF	0.29	0.057	12.76	(30/30) 100%	(0/30) 0%
Low Positive Serum	0.25	0.036	14.40	(29/30) 96.7%	(1/30) 3.3%
High Negative BALF	0.17	0.104	Not Applicable	(28/30) 93.3%	(2/30) 6.7%
Negative BALF	0.03	0.056	Not Applicable	(0/30) 0%	(30/30) 100%
Negative Serum	0.06	0.043	Not Applicable	(0/30) 0%	(30/30) 100%

### 3.6 Interference and cross-reactivity

The influence of endogenous serum components known to cause matrix interference (hemoglobin, bilirubin, triglyceride) was investigated. Spiking experiments showed no measurable interference effect in the assay system. Concurrently, the potential for cross-reactivity of the CLEIA was also examined using a diverse set of patient serum samples collected from individuals with clinically relevant conditions. Results of cross-reactivity testing are compiled in **Table 3**. A *Histoplasma* serum specimen was found positive by the present chemiluminescent immunoassay. Notably, the single *Histoplasma* cross-reactive serum specimen was also positive on another commercially available Platelia GM-ELISA, with a GM Index of 4.20, compared to 0.36 CLEIA Units on the CLEIA.

**Table 3:** The effect of potentially interfering medical conditions unrelated to IPA.

Pathogen	Number of specimens	% Positive
Influenza A virus	15	0% (0/15)
SARS-CoV-2	12	0% (0/12)
*Mycoplasma*	8	0% (0/8)
*Staphylococcus aureus*	5	0% (0/5)
*Mycobacterium tuberculosis*	10	0% (0/10)
*Blastomyces dermatitidis*	5	0% (0/5)
*Candida albicans*	5	0% (0/5)
*Cryptococcus neoformans*	10	0% (0/10)
*Histoplasma capsulatum*	6	16.7% (1/6)
*Penicillium chrysogenum*	10	0% (0/10)
*Fusarium solani* sp*ecies complex*	3	0% (0/3)
*Scedosperium apiospermum*	5	0% (0/5)

### 3.7 Method comparison

**Figure 4:**
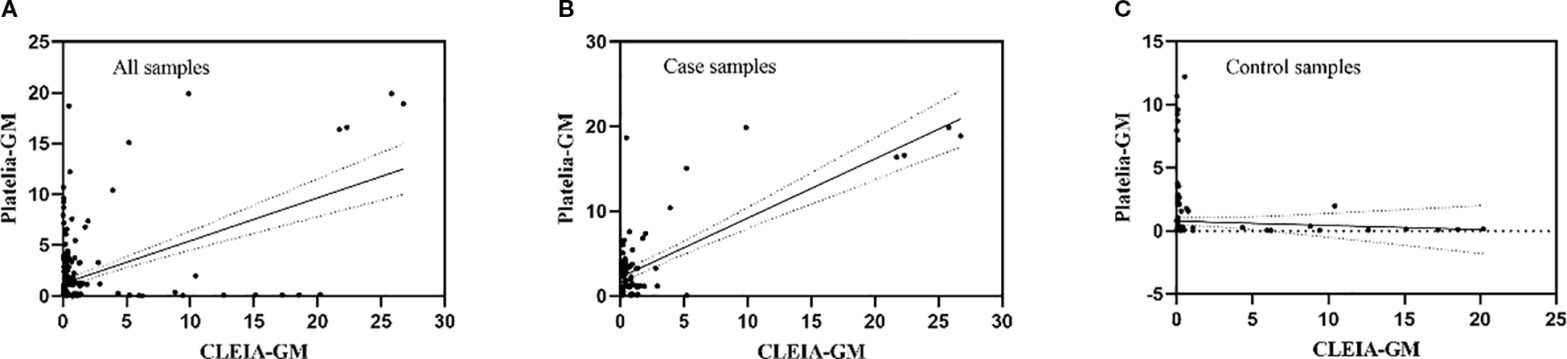
Correlation analysis of candidate CLEIA versus commercial Platelia ELISA kit. **(A)** All samples. **(B)** Cases only. **(C)** Controls only. The solid black line represents the linear regression fit; dot-dash lines indicate the 95% confidence bands.

A cohort of 241 specimens (86 cases, 155 controls) underwent parallel testing with both the FDA-approved Platelia GM ELISA test and the developed chemiluminescent immunoassay. Qualitative assessment between platforms showed high diagnostic concordance across sample cohorts: percent positive agreement = 71.68% (95% CI, 62.30–79.56%*)*, percent negative agreement = 80.00% (95% CI, 72.79–85.73%), and overall agreement = 86.72% (95% CI, 81.63–90.61%). This corresponded to a Cohen’s kappa statistic of 0.72 (95% CI, 0.64–0.81), indicative of substantial agreement according to the Landis and Koch scale (**Table 4**) (Landis and Koch, 1977). The overall quantitative correlation between the GM index values calculated by the candidate CLEIA and the Platelia ELISA was moderate (Spearman’s ρ = 0.48, *P <*0.0001; **Figure 4A**). Significantly stronger agreement was observed in case-derived specimens (ρ = 0.75, P < 0.0001; **Figure 4B**), while control samples showed no statistically significant correlation (ρ = -0.05, *P*=0.50; **Figure 4C**).

**Table 4:** Concordance rate between CLEIA and Platelia ELISA.

No. with ^b^CLEIA result of	^a^Platelia ELISA		Total
	Positive	Negative	
Positive	81	20	101
Negative	12	128	140
Total	93	148	241
Positive agreement (%) (95% CI)	71.68% (62.30–79.56%)		
Negative agreement (%) (95% CI)	80.00% (72.79–85.73%)		
Overall agreement (%) (95% CI)	86.72% (81.63–90.61%)		
κ (CI 95%)	0.72 (0.64–0.81)		

a Platelia ELISA: positivity threshold, GM index ≥1.0 in serum or BALF.

b CLEIA: positivity threshold, GM CLEIA Units ≥ 0.2 in serum or BALF.

## 4 Discussion

We developed a rapid, high-sensitive and selective GM immunoassay leveraging magnetic separation technology and ALP-APS chemiluminescence. This platform demonstrates four key advancements over conventional methodologies. Firstly, antibody-functionalized magnetic beads ensure homogeneous antigen capture in solution, while integrated magnetic rod technology automates critical workflow steps including incubation and washing. This dual-approach eliminates manual intervention requirements characteristic of ELISA and lateral flow assays (LFA), enabling true high-throughput screening scalability (Gallet et al., 2021). Secondly, implementation of a streamlined single-incubation protocol reduces total analysis time to 30min (including a 20-min antigen-antibody binding phase) - representing a 75% reduction versus the 120-min Platelia GM gold standard, while maintaining equivalent diagnostic accuracy. Thirdly, photomultiplier-based quantification of amplified chemiluminescent signals provides significantly higher analytical sensitivity than spectrophotometric chromogen detection in ELISA. Clinical validation confirmed 9.30% greater diagnostic yield (78/86 vs.70/86 in clinically suspected specimens) without compromising specificity. Lastly, this assay demonstrates strong clinical utility, delivering timely and reliable results. Its capacity for single-sample processing makes it particularly suitable for urgent specimens, eliminating the need for batch testing or sample accumulation. Each independent test provides same-day results, optimizing workflow efficiency in critical care settings (Calero et al., 2022).

Detection of GM antigen, primarily using the Platelia ELISA, offers an alternative direct diagnostic method applicable to various samples, including BALF and serum. While initial performance studies of the Platelia ELISA suggested high sensitivity (70–85%) (Leeflang et al., 2015), a recent work by Karl et al. reported significantly lower sensitivity—53% in patients with hematologic malignancies and 33% in those without (Dichtl et al., 2019). Consequently, the author caution against relying solely on GM antigen testing for diagnosing IPA, particularly in non-hematologic populations. Although the CLEIA developed in this study demonstrated excellent qualitative agreement with the Platelia ELISA, further validation in larger cohorts of confirmed IPA patients is essential to robustly define its clinical sensitivity across diverse disease manifestations associated with *Aspergillus* infection.

CLEIA have gained attraction in recent years for their rapid results and automation. Literature reports GM CLEIA sensitivities ranging from 60% to 85% (Calero et al., 2022; Buil et al., 2023; Schub et al., 2024). In a study by Troncoso et al., the Platelia *Aspergillus* Ag ELISA was compared with an *Aspergillus* GM CLEIA assay. Both methods demonstrated 85.7% agreement in serum samples. The GM CLEIA showed approximately 16% higher sensitivity than the Platelia ELISA, whereas the latter exhibited 11% greater specificity (Troncoso et al., 2022). In our study, the higher sensitivity (90.70%) of GM CLEIA might be attributed to the smaller number of patients diagnosed with IPA. While the retrospective case-control design at a single center inherently limits generalizability (potentially explaining performance differences), robust validation awaits prospective multicenter studies.

In this study, we identified ≥0.20 CLEIA Units as the optimal cutoff for positivity in both serum and BALF samples. However, reported diagnostic optical density index (ODI) cutoffs for GM ELISA vary considerably across studies and patient populations (Ambasta et al., 2015). Additionally, the absence of an established equivocal zone (“grey zone”) necessitates cautious interpretation of values near this cutoff. Specifically, such borderline results require supportive mycological, radiological, or clinical evidence of *Aspergillus* infection. Finally, since not all samples from confirmed cases yield positive results, definitive diagnosis demands comprehensive multidimensional assessment.

The commercial Platelia assay employs a single rat monoclonal antibody (EB-A2) targeting β-(1,5)-linked galactofuranoside side chains of GM (Stynen et al., 1992; Stynen et al., 1995). Due to structural similarities in β-glucans among fungi, this assay exhibits cross-reactivity with GM from other invasive fungi like *Penicillium, Histoplasma capsulatum*, and *Candida* sp*ecies* (Cummings et al., 2007; Wheat et al., 2007; Tortorano et al., 2012). In contrast, the newly developed GM CLEIA method incorporates two distinct antibody types, potentially enhancing specificity. However, because these antibodies likely recognize epitopes similar to EB-A2, cross-reactivity with GM from fungi such as *Histoplasma capsulatum* is still observed. Given the potential for false positives from various factors, extensive cross-reactivity studies remain essential for clinical validation of this new method.

Several limitations merit acknowledgment, most notably the sole inclusion of unique patients and lack of a clinical outcome-based correlation such as mortality or timing of antifungal initiation, restricting this detection method’s current utility to clinical diagnosis. We did not assess GM antigen trends over time in serial patient samples– though this represents an active focus of our current research. Consequently, we plan a comprehensive validation study in a larger, more diverse population, encompassing varying epidemiological contexts and timepoints from symptom onset. In addition, this study was performed with banked samples that were tested after they were stored and frozen, although it is unlikely that this had a relevant impact on test performance (Prattes et al., 2015; Gallet et al., 2021; Egger et al., 2022). Furthermore, we did not perform cost analysis or assess the economic feasibility of implementing CLEIA in routine diagnostics.

## 5 Conclusions

Despite the limitations outlined here, our data support the use of the developed CLEIA as a viable alternative method for qualitative detection of GM antigen in serum and BALF. Full automation of the CLEIA system would render it considerably more applicable than the current implementation. This work provides valuable insights into the clinical applicability of CLEIA testing, paving the way for larger-scale validation studies required to substantiate these findings and improve their diagnostic reliability.

## Data Availability

The original contributions presented in the study are included in the article/supplementary material. Further inquiries can be directed to the corresponding author/s.

## References

[B1] Albert E. Alcaraz M. J. Giménez E. Clari M. Á. Torres I. Colomina J.. (2024). Comparative performance of the Platelia *Aspergillus* Antigen and *Aspergillus* Galactomannan antigen Virclia Monotest immunoassays in serum and lower respiratory tract specimens: a “real-life” experience. Microbiol. Spectr. 12, e0391023. doi: 10.1128/spectrum.03910-23, PMID: 38916338 PMC11302238

[B2] Ambasta A. Carson J. Church D. L. (2015). The use of biomarkers and molecular methods for the earlier diagnosis of invasive aspergillosis in immunocompromised patients. Med. Mycol. 53, 531–557. doi: 10.1093/mmy/myv026, PMID: 26026174

[B3] Arastehfar A. Carvalho A. Houbraken J. Lombardi L. Garcia-Rubio R. Jenks J. D.. (2021). Aspergillus fumigatus and aspergillosis: From basics to clinics. Stud. Mycol. 100, 100115. doi: 10.1016/j.simyco.2021.100115, PMID: 34035866 PMC8131930

[B4] (2022). WHO fungal priority pathogens list to guide research, development and public health action (Geneva: World Health Organization). Licence: CC BY-NC-SA 3.0 IGO.

[B5] Buil J. B. Huygens S. Dunbar A. Schauwvlieghe A. Reynders M. Langerak D.. (2023). Retrospective multicenter evaluation of the virClia galactomannan antigen assay for the diagnosis of pulmonary Aspergillosis with bronchoalveolar lavage fluid samples from patients with hematological disease. J. Clin. Microbiol. 61, e0004423. doi: 10.1128/jcm.00044-23, PMID: 37097150 PMC10204623

[B6] Calero A. L. Alonso R. Gadea I. Vega M. D. M. García M. M. Muñoz P.. (2022). Comparison of the performance of two galactomannan detection tests: Platelia aspergillus Ag and Aspergillus galactomannan ag virclia monotest. Microbiol. Spectr. 10, e0262621. doi: 10.1128/spectrum.02626-21, PMID: 35262395 PMC9045373

[B7] Cummings J. R. Jamison G. R. Boudreaux J. W. Howles M. J. Walsh T. J. Hayden R. T. (2007). Cross-reactivity of non-Aspergillus fungal species in the Aspergillus galactomannan enzyme immunoassay. Diagn. Microbiol. Infect. Dis. 59, 113–115. doi: 10.1016/j.diagmicrobio.2007.04.022, PMID: 17662550

[B8] Denning D. W. (2024). Global incidence and mortality of severe fungal disease. Lancet Infect. Dis. 24, e428–e438. doi: 10.1016/S1473-3099(23)00692-8, PMID: 38224705

[B9] Dichtl K. Seybold U. Ormanns S. Horns H. Wagener J. (2019). Evaluation of a novel Aspergillus antigen enzyme-linked immunosorbent assay. J. Clin. Microbiol. 57, e00136–e00119. doi: 10.1128/JCM.00136-19, PMID: 31018980 PMC6595454

[B10] Donnelly J. P. Chen S. C. Kauffman C. A. Steinbach W. J. Baddley J. W. Verweij P. E.. (2020). Revision and update of the consensus definitions of invasive fungal disease from the European organization for research and treatment of cancer and the mycoses study group education and research consortium. Clin. Infect. Dis. 71, 1367–1376. doi: 10.1093/cid/ciz1008, PMID: 31802125 PMC7486838

[B11] Egger M. Penziner S. Dichtl K. Gornicec M. Kriegl L. Krause R.. (2022). Performance of the euroimmun Aspergillus antigen ELISA for the diagnosis of invasive pulmonary aspergillosis in bronchoalveolar lavage fluid. J. Clin. Microbiol. 60, e0021522. doi: 10.1128/jcm.00215-22, PMID: 35350844 PMC9020356

[B12] Fröhlich-Nowoisky J. Pickersgill D. A. Després V. R. Pöschl U. (2009). High diversity of fungi in air particulate matter. Proc. Natl. Acad. Sci. U.S.A. 106, 12814–12819. doi: 10.1073/pnas.0811003106, PMID: 19617562 PMC2722276

[B13] Gallet S. Garnaud C. Dragonetti C. Rivoiron S. Cognet O. Guo Y.. (2021). Evaluation of a prototype of a novel galactomannan sandwich assay using the VIDAS^®^ Technology for the diagnosis of invasive aspergillosis. Front. Cell Infect. Microbiol. 11. doi: 10.3389/fcimb.2021.669237, PMID: 34336710 PMC8322699

[B14] Hajjeh R. A. Warnock D. W. (2001). Counterpoint: invasive aspergillosis and the environment–rethinking our approach to prevention. Clin. Infect. Dis. 33, 1549–1552. doi: 10.1086/322970, PMID: 11568854

[B15] Herrera S. Magyar U. Husain S. (2025). Invasive aspergillosis in the current era. Infect. Dis. Clin. North Am. 39, e33–e60. doi: 10.1016/j.idc.2025.01.002, PMID: 40157842

[B16] Kılıç E. Şahin E. A. Tunçcan Ö. G. Yıldız Ş. Özkurt Z. N. Yeğin Z. A.. (2025). Comparative analysis of chemiluminescence immunoassay (CLIA)-based tests in the diagnosis of invasive Aspergillosis in patients with hematologic Malignancies. Mycoses 68, e70064. doi: 10.1111/myc.70064, PMID: 40277032 PMC12023017

[B17] Landis J. R. Koch G. G. (1977). The measurement of observer agreement for categorical data. Biometrics 33, 159–174. doi: 10.2307/2529310 843571

[B18] Leeflang M. M. Debets-Ossenkopp Y. J. Wang J. Visser C. E. Scholten R. J. Hooft L.. (2015). Galactomannan detection for invasive aspergillosis in immunocompromised patients. Cochrane Database Syst. Rev. 2015, CD007394. doi: 10.1002/14651858.CD007394.pub2, PMID: 26716951 PMC6483812

[B19] Lo Cascio G. Lepera V. Sorrentino A. Caleca D. Gigante P. Tocci G.. (2024). Evaluation of a new automated mono-test for the detection of *Aspergillus* galactomannan: comparison of *Aspergillus* galactomannan ag virCLIA^®^ Mono-test with platelia™*Aspergillus* ag ELISA assay. J. Fungi (Basel). 10, 793. doi: 10.3390/jof10110793, PMID: 39590712 PMC11595404

[B20] Mercier T. Guldentops E. Lagrou K. Maertens J. (2018). Galactomannan, a surrogate marker for outcome in invasive aspergillosis: finally coming of age. Front. Microbiol. 9. doi: 10.3389/fmicb.2018.00661, PMID: 29670608 PMC5893815

[B21] Miceli M. H. Kauffman C. A. (2017). Aspergillus galactomannan for diagnosing invasive aspergillosis. JAMA 318, 1175–1176. doi: 10.1001/jama.2017.10661, PMID: 28973595

[B22] Moura S. Cerqueira L. Almeida A. (2018). Invasive pulmonary aspergillosis: current diagnostic methodologies and a new molecular approach. Eur. J. Clin. Microbiol. Infect. Dis. 37, 1393–1403. doi: 10.1007/s10096-018-3251-5, PMID: 29754210

[B23] Ostrosky-Zeichner L. (2012). Invasive mycoses: diagnostic challenges. Am. J. Med. 125, S14–S24. doi: 10.1016/j.amjmed.2011.10.008, PMID: 22196205

[B24] Prattes J. Koidl C. Eigl S. Krause R. Hoenigl M. (2015). Bronchoalveolar lavage fluid sample pretreatment with Sputasol(^®^) significantly reduces galactomannan levels. J. Infect. 70, 541–543. doi: 10.1016/j.jinf.2014.11.005, PMID: 25447710

[B25] Schub T. Klugherz I. Wagener J. Prattes J. Hoenigl M. Suerbaum S.. (2024). Serum antigen tests for the diagnosis of invasive Aspergillosis: a retrospective comparison of five Aspergillus antigen assays and one beta-D-glucan assay. J. Clin. Microbiol. 62, e0095024. doi: 10.1128/jcm.00950-24, PMID: 39494863 PMC11633112

[B26] Schwarz M. C. R. Moskaluk A. E. Daniels J. B. VandeWoude S. Reynolds M. M. (2024). Current analytical methods and challenges for the clinical diagnosis of invasive pulmonary aspergillosis infection. J. Fungi (Basel). 10, 829. doi: 10.3390/jof10120829, PMID: 39728325 PMC11676737

[B27] Stynen D. Sarfati J. Goris A. Prévost M. C. Lesourd M. Kamphuis H. (1995). A new sensitive sandwich enzyme-linked immunosorbent assay to detect galactofuran in patients with invasive aspergillosis. J. Clin. Microbiol. 33, 497–500. doi: 10.1128/jcm.33.2.497-500.1995, PMID: 7714217 PMC227977

[B28] Stynen D. Sarfati J. Goris A. Prévost M. C. Lesourd M. Kamphuis H.. (1992). Rat monoclonal antibodies against Aspergillus galactomannan. Infect. Immun. 60, 2237–2245. doi: 10.1128/iai.60.6.2237-2245.1992, PMID: 1375195 PMC257149

[B29] Terrero-Salcedo D. Powers-Fletcher M. V. (2020). Updates in laboratory diagnostics for invasive fungal infections. J. Clin. Microbiol. 58, e01487–e01419. doi: 10.1128/JCM.01487-19, PMID: 32132194 PMC7269377

[B30] Tortorano A. M. Esposto M. C. Prigitano A. Grancini A. Ossi C. Cavanna C.. (2012). Cross-reactivity of Fusarium spp. in the Aspergillus Galactomannan enzyme-linked immunosorbent assay. J. Clin. Microbiol. 50, 1051–1053. doi: 10.1128/JCM.05946-11, PMID: 22205818 PMC3295092

[B31] Troncoso C. R. Sepúlveda F. C. Sepúlveda P. E. Guzmán U. C. Morales G. M. Tapia P. C.. (2022). Evaluation of the Aspergillus Galactomannan ag VircliaR Monotest test as an alternative to Platelia™ Aspergillus EIA kit. Rev. Chil. Infectol. 39, 248–253. doi: 10.4067/s0716-10182022000200248, PMID: 36156685

[B32] Wheat L. J. Hackett E. Durkin M. Connolly P. Petraitiene R. Walsh T. J.. (2007). Histoplasmosis-associated cross-reactivity in the BioRad Platelia Aspergillus enzyme immunoassay. Clin. Vaccine Immunol. 14, 638–640. doi: 10.1128/CVI.00479-06, PMID: 17344352 PMC1865624

